# Welch's Rubber Solder

**Published:** 1870-11

**Authors:** 


*Welch's Rubber Solder.*—This is a new preparation for repairing vulcanite work, and in the several cases in which we have tried it, answered the the purpose admirably, although sufficient time has not elapsed to test its durability.

Benzine appears to be the solvent, and it evidently owes its efficacy in a great measure to the presence of this agent. The following directions are given for the application of this preparation: "Invest the case in plaster, leaving the part exposed to which it is desired that the new rubber shall adhere. Scrape this surface clean, and with a camels hair brush coat with the liquid, allowing it partly to evaporate; then apply new rubber slightly warmed, press the parts of the flask together, and vulcanize." It is claimed that the union will be as strong as any other part of the work.

